# Analytical solution of the classical Rayleigh length definition, including truncation at arbitrary values

**DOI:** 10.1111/jmi.70016

**Published:** 2025-07-31

**Authors:** Aufried Lenferink, Cees Otto

**Affiliations:** ^1^ Department of Bio Engineering and Technology, Technical Medical Centre, Faculty of Science & Technology University of Twente Enschede The Netherlands

**Keywords:** diffraction integral, Gaussian beam, microscopy, optical resolution, Rayleigh length, truncation

## Abstract

We present the analytical solution to the diffraction integral that describes the Rayleigh length for a focused Gaussian beam with any value of a spherical truncating aperture. This exact solution is in precise agreement with numerical calculations for the light distribution in the near focal area. The solution arises under assumption of the paraxial approximation, which also provides the basis for the classical Rayleigh length definition. It will be shown that the non‐paraxial regime can be included by adding an empirical term (*C*_np_) to the solution of the diffraction integral. This extends the validity of the expression to high numerical apertures (*NA*) up to *n* times 0.95, with *n* being the refractive index of the immersion medium. Thus, the entire practical range of NA, encountered in optical microscopy, is covered with a calculated error of less than 0.4% in the non‐paraxial limit. This theoretical result is important in the design of optical instrumentation, where overall light efficiency in excitation and detection and spatial resolution must be optimised together.

## LAY DESCRIPTION

The development and construction of high‐resolution optical microscopes for applications in which lasers with Gaussian beams are used to generate the smallest possible focal spot, such as in Raman microscopy or single‐molecule fluorescence microscopy, requires a careful balance between the design parameters: laser beam truncation, light power and spatial resolution. Optimisation of one property will come at the expense of another property and all parameters are critical in the microscope design. The theoretical description of the effect of all parameters on the quantitative performance of an optical microscope requires the solution of a diffraction integral, which contains all these parameters. The diffraction integral describes the intensity distribution along the optical axis in the focal region, as a function of these parameters. The analytical solution of this diffraction integral has not previously been shown and includes the truncation parameter from first principles. For an untruncated Gaussian beam the well‐known definition of the Rayleigh length is obtained. The new theory connects theoretical considerations directly with practical implementation which is valuable for the optimisation of a microscope design. The theoretical expression presented here can be seen as an expansion of Gaussian beam theory for practical and general applications. The analytical solution is a paraxial expression and can be extended to the non‐paraxial regime of high NA objectives with a small error of 0.4%. The presented theory is therefore applicable to the entire range of numerical aperture encountered in practical optical microscopy.

## 1 INTRODUCTION

Accurate knowledge of the spatial properties of the focal field is important to optimise the design of microscope measurement systems for high resolution optical microscopy, such as fluorescence—and Raman microscopy. Not only will an optimally focused beam enable the highest resolution, it will also enable the highest signal‐to‐background ratio and, consequentially, the highest signal to noise ratio. This becomes increasingly important when objects must be detected that are much smaller than the focal spot dimensions, as is commonly the case in the detection and characterisation of biological nano‐particles, such as extracellular vesicles and viruses,^1^ or non‐biological particles, such as nano‐plastics in water.^2^

Laser beams often have a Gaussian (TEM00) intensity distribution. To effectively make use of the available laser power, the degree of truncation of this Gaussian beam at the entrance pupil of the focusing element has to be considered. To attain the required spatial resolution and detection efficiency, high *NA* optics are preferred. However, when the radius of the laser beam is smaller than the radius of the entrance pupil, the *NA* is not completely used and focal spot dimensions are increased. Alternatively, when the radius of the laser beam is larger than the entrance pupil, the intensity distribution in the focus will tend towards an Airy pattern with a small focal spot but with inefficient use of available laser power. It is clear that an optimisation of light‐loss versus resolving‐power is an integral aspect of the design of a microscope. This aspect has been considered in detail in the literature,^3^, ^4^, ^5^, ^6^, ^7^, ^8^, ^9^, ^10^ where mathematical expressions involve unsolved integrals, approximations, limitations in Truncation‐range and/or empirical terms with well‐chosen, but arbitrary constants. Mahajan treated the properties of the focused field in terms of Gauss‐Zernike polynomials and showed results for discrete values of the truncation.^11^ Here, we present the paraxial analytical expression for the on‐axis intensity distribution including the truncation value. To include the range of high NA objectives, it will be shown that the paraxial analytical expression can be extended with an empirical term to capture the non‐paraxial region as well. The results are in full agreement with the outcome of numerical calculations of the exact diffraction integral. The exact diffraction integral is described in detail and presented by Equation (15).

## 2 GAUSSIAN BEAM THEORY

The Rayleigh length $Z_{r}$ is the distance along the propagation direction over which the peak irradiance of the beam has dropped by a factor 2 with respect to the irradiance in the focal plane. It therefore defines the longitudinal size of the focal region. For a Gaussian beam, propagating along the axis *z*, the intensity distribution is described as follows:^12^

(1) $$\begin{array}{ccc} I\;\left(r,z\right) & = & I_{0}\;\frac{{W_{0}}^{2}}{W^{2}\left(z\right)}\exp\left[-\frac{2r^{2}}{W^{2}\left(z\right)}\right],W^{2}\;\left(z\right)\\ & = & W_{0}^{2}\;\left[1+\frac{z^{2}}{Z_{r}^{2}}\right]. \end{array}$$

**FIGURE 1:**
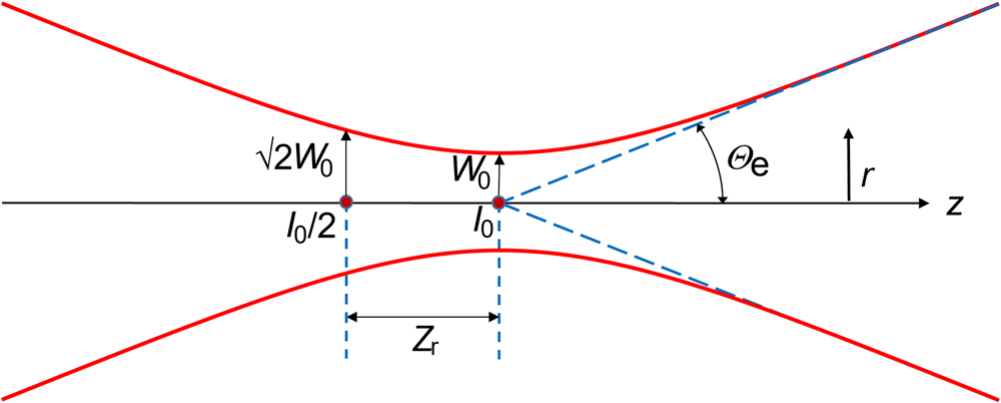
Intensity contour (1/*e* ^2^) of a focused Gaussian beam.

A graph of this function is presented in Figure 1.

The red contour in Figure 1 is the $1/e^{2}$ intensity level. At the centre of the waist, the intensity is *I*_0_. When displacing from the centre by distance $Z_{r}$ the radius increases by a factor of √2. The Rayleigh length $Z_{r}$ is given by:^13^, ^14^

(2) $$Z_{r}=\frac{\pi nW_{0}^{2}}{\lambda_{0}},$$

where $W_{0}$ is the minimum beam radius at z=0, $\lambda_{0}$ is the vacuum wavelength and *n* is the refractive index of the (immersion) medium in which the focus is formed. With the use of Equation (1), the cone angle $\theta_{e}\;$ can be derived:

(3) $$\tan\;\left(\Theta_{e}\right)=\frac{W_{0}}{Z_{r}},$$

where the subscript ‘*e*’ signifies that it is associated with the numerical aperture of the $1/e^{2}$ ‐light cone.

Thus, the waist radius $W_{0}$ and $Z_{r}$ can also be defined as

(4) $$W_{0}=\frac{\lambda_{0}}{\pi NA_{e}},$$

(5) $$Z_{r}=\frac{n\lambda_{0}}{\pi NA_{e}^{2}},$$

with $NA_{e}=n\sin\left(\theta_{e}\right)\approx n\tan\left(\theta_{e}\right)$. These expressions of $Z_{r}$ and $W_{0}$ are commonly used to define the optical axial‐ and lateral‐resolution. In the expression of $W_{0}$ and $Z_{r}$ an approximation is used, namely, that sinθ≈tanθ. The expression (Equation 5) for the Rayleigh range is therefore an approximation that is only valid for paraxial applications and for non‐truncated Gaussian beams.

Here, we will show a more general expression for the Rayleigh range, which incorporates the numerical aperture after truncation, called $NA_{T}$, and that can be extended to include high numerical aperture optics, where the paraxial limit does not hold anymore. The Gaussian beam truncation takes place at some field stop in the set up. Without loss of generality, we have chosen this to be the diameter of the entrance pupil of the focusing element.

## 3 BEAM TRUNCATION EFFECT

The diameter of the physical focusing element or its pupil diameter acts as the beam‐truncating aperture in a microscope. The degree of truncation is defined by the parameter *T* and forms the ratio of the radius of the $1/e^{2}$‐contour of the Gaussian beam *(R*_e_) at the entrance pupil and the radius of the entrance pupil itself ($R_{T}$):

(6) $$T=\frac{R_{e}}{R_{T}}.\;$$

**FIGURE 2:**
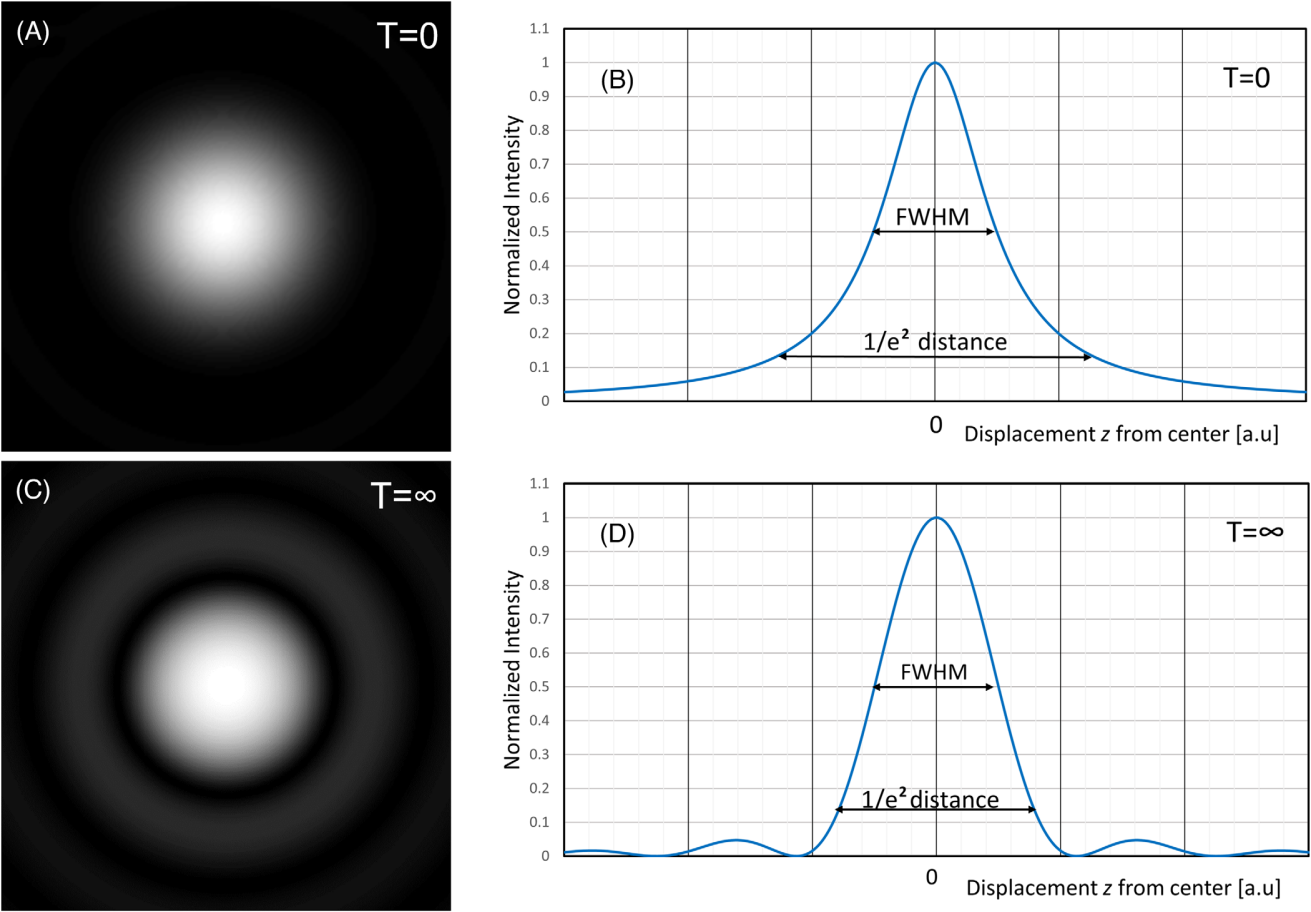
Intensity distribution in the focal area. (A, C) Intensity distribution in the lateral plane (*z* = 0). (B, D) Intensity profile along the optical axis (*r* = 0) for a Gaussian illumination (A and D) and a planar field illumination (C and D).

Truncation not only lead to a power‐loss, but it also leads to a change in the otherwise Gaussian shaped focal spot after the truncating element, and affects the size of $Z_{r}$ and $W_{0}$, which describe the amplitude distribution around the focal plane through Equation (1). The truncation parameter varies from T=0 to T=∞. The lateral profile of the focal spot for a Gaussian beam distribution is shown in Figure 2A and B and for an Airy beam distribution is shown in Figure 2C and D.

The power loss due to truncation can be derived from Equation (1) and is given by:^15^

(7) $$P_{\mathrm{loss}}=\exp\left[-\frac{2}{T^{2}}\right].$$

As mentioned in Section 2, Equations (4) and (5) are only valid in the paraxial region, hence at low *NA*. In high‐resolution microscopy and at low signal photon flux, the use of high *NA* optics is to be preferred. In addition, biological objects are usually embedded in water as a medium, with a refractive index, *n*, of ∼1.33, and observed below a coverslip with an objective with a cover‐glass correction to reduce astigmatism. Therefore, we have consistently included the dependency on the refractive index, *n*, in this work.

**FIGURE 3:**
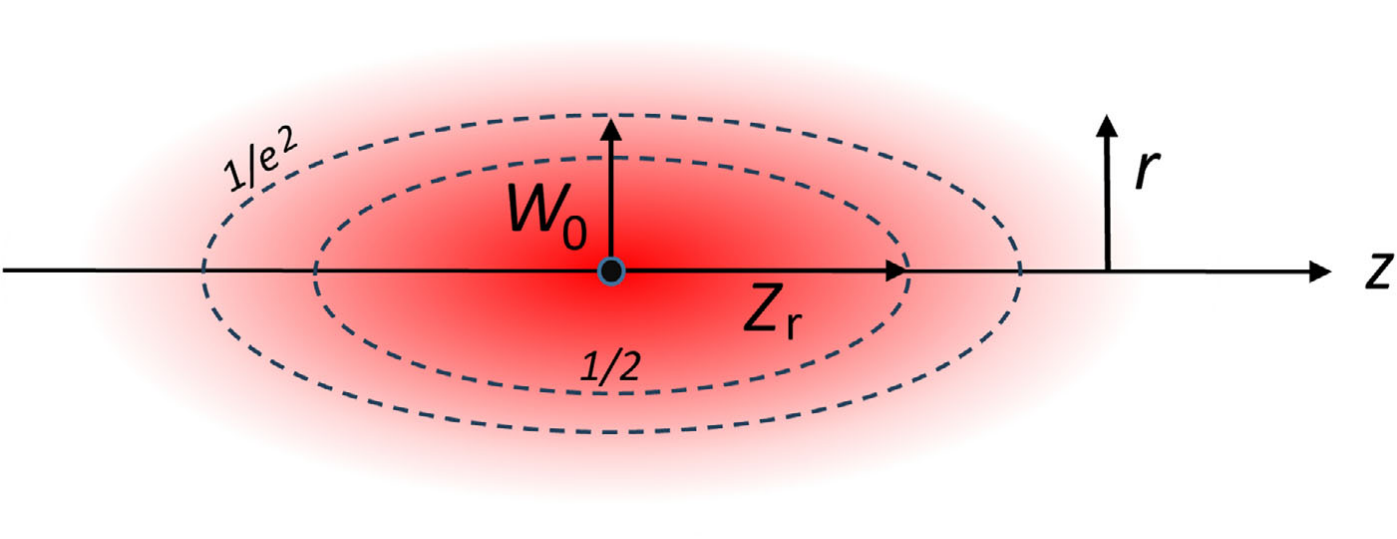
An impression of the focal area and the intensity contours corresponding with the definitions of the Rayleigh length *Z*_r_ and *W*_o_.

As we noted, the Rayleigh range $Z_{r}$ in Equation (5) is based on a reduction of light intensity by a factor of 2 along the propagation direction and the waist of the beam $W_{0}$ in Equation (4) is based on a $1/e^{2}$ intensity definition in the radial direction thus the definitions are therefore based on different intensity criteria of the beam. It is more logical to express parameters for both dimensions of the beam based on the same criterion for the intensity. The use of the same intensity criteria also leads to a uniform definition of a focal volume, as shown in Figure 3.

To achieve this, a normalised intensity profile $I_{zn}$ and $I_{rn}$ along the optical axis is introduced, instead of only a $Z_{r}$ value at the FWHM ($I_{\mathrm{zn}}$= 1/2) condition and $W_{0}$ value at the $I_{rn}$=$1/e^{2}\;$ condition. When available, a resolution distance *r* and *z* can be found for any relative intensity level $I_{\mathrm{rn}}=I_{\mathrm{zn}}\;$ chosen. This will make it possible to determine the focal spot volume by assuming it is an ellipsoid with axis r and z. In general $I_{\mathrm{zn}}$ and $I_{\mathrm{rn}}$ are of the form $I_{\mathrm{zn}}=f\left(z,T,NA_{T},\lambda_{0},n\right)$ and $I_{\mathrm{rn}}=f\left(r,T,NA_{T},\lambda_{0},n\right)$, with $NA_{T}$, the numerical aperture of the optical system. Instead of using the numerical aperture $\;NA_{T}$, the f‐number *F#*_T_ of the truncated beam will be used, defined by *F#*_T_ *= f*/2*R*_T_ and *F#*_e_ *= f*/2*R*_e_, with *f* the focal distance. A rational for this is that, since the *NA* is defined as NA=nsinθ and no valid outcome is obtained for angles $\theta >90^{\circ }$, the f‐number *F#*_T_ does not have this limitation and the angles for rays towards the focal point can now acquire values between $0^{\circ }$ and $180^{\circ }$. The convenience of this is further explained in Section 4. The definition for $Z_{r}$ (Equation 5) in terms of *F#*_T_ then becomes:

(8) $$Z_{r}=\frac{4\lambda_{o}F{{\#}_{e}}^{2}}{\pi \;n}\;=\frac{4\lambda_{o}\cdot F{{\#}_{T}}^{2}}{\pi \;nT^{2}}.$$

To convert *F*# to *NA* and vice versa the following relations are valid, also outside the paraxial region:

(9)

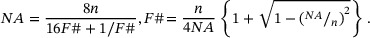




These relations are identical, based on the definitions $NA=n\sin\theta \;\mathrm{and}\;F\#=\frac{f}{2r}\;$ and can be derived when considering a parabolic interface as described in the next section.

## 4 PARABOLIC MIRROR MODEL

**FIGURE 4:**
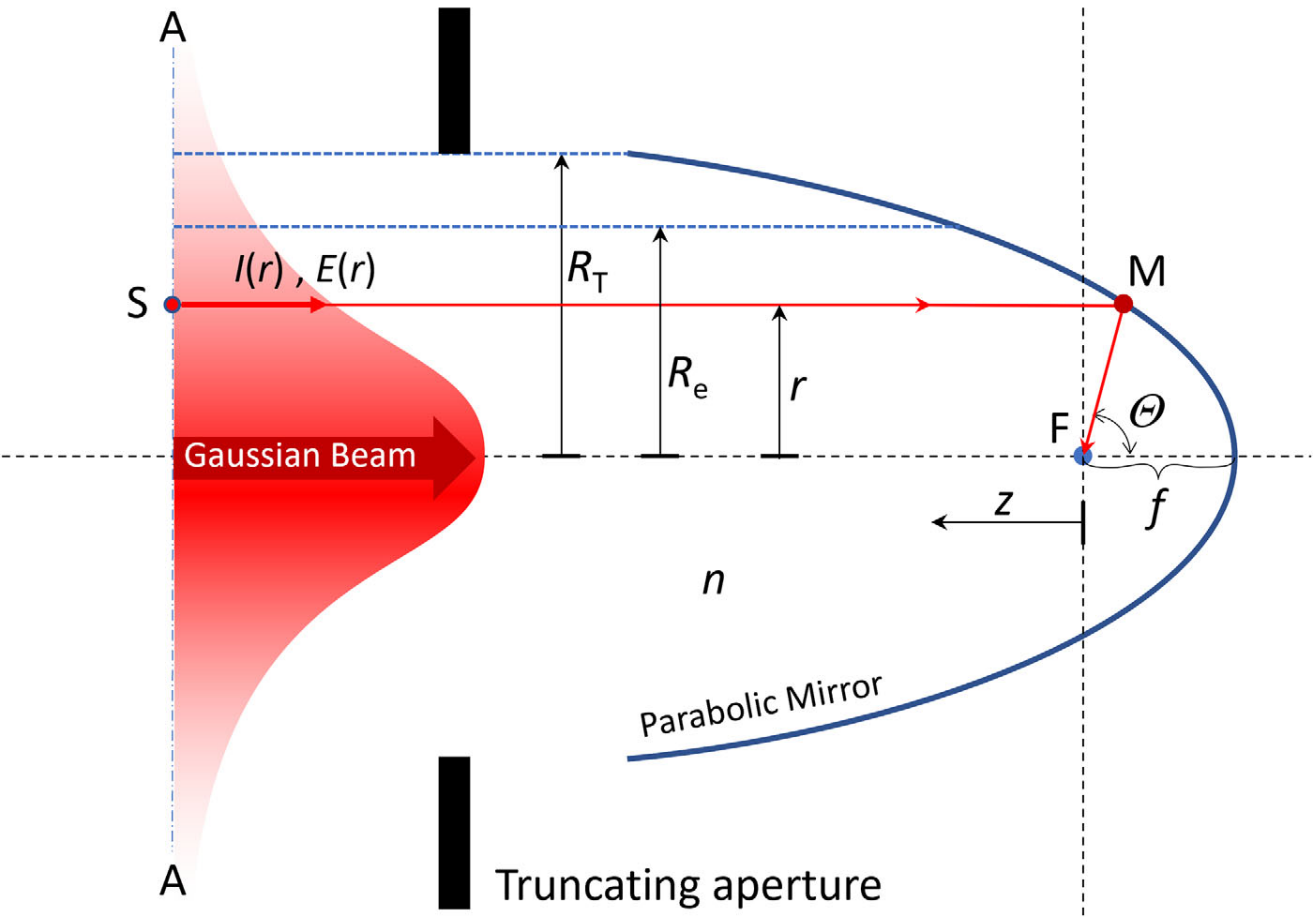
Parabolic mirror as the focusing element.

Here we introduce a parabolic mirror as a model to produce a focus. The parabolic mirror is a focusing element with a single interface that is capable of directing all incoming rays parallel to the optical axis towards a single point, as shown in Figure 4. The use of a parabolic mirror as a focusing element is not only widely used in astronomy by radio antennas and optical telescopes, but it is also sometimes implemented in high NA microscopy objectives.^16^ Although the parabolic mirror is the perfect focusing element for creating the smallest focal spot for all wavelengths at the same time on the optical axis, it suffers from astigmatism and other errors when focusing off‐axis. This is the reason why high NA objectives are made of multiple lenses to compensate for lens errors. However for describing the perfect focus intensity distribution on the optical axis, we can conceptually use the parabolic mirror model, as follows.

A parabolic mirror captures all angles, $0^{\circ }<\theta <180^{\circ }$ as long as the mirror is large enough. The parabolic mirror directs all parallel incoming rays along the optical axis towards a single point F, which is the focus of the parabolic mirror. The optical path length for all rays parallel to the optical axis from any phase front, for example, indicated by A‐A, perpendicular to the optical axis (z‐axis) and the focal point, is equal and therefore will reach the focal point F with the same phase and thus constructively interfere to the highest intensity at F. Truncation, *T*, can be incorporated by considering only the incoming rays below the truncation radius *R*_T_. The focal point F is located at coordinates (r,z)=(0,0). Any point with *r*≠0 and/or *z*≠0 will experience phase differences between incoming rays, which will result in a lower intensity at that point. By applying the Huygens‐Fresnel principle and mapping the Gaussian intensity distribution over the parabolic mirror surface, we can consider it as a surface with sources. The phase differences between these source locations on this parabolic surface is determined by the distance of plane A‐A to the mirror surface at M, that is, the distance SM. The total phase difference for a ray reaching a point on the z‐axis at *z* is determined by the total distance SM*z*. This distance *L* can be described with:

(10) $$L=L_{0}+z\;cos\left(\Theta \right),\;cos\;\left(\Theta \right)=\frac{4f^{2}-r^{2}}{4f^{2}+r^{2}}.\;$$

This relation is valid as long as *z<<f*. This complies with the present situation, where f is of the order of millimetre and *z* is of the order of micrometre. $L_{0}$ is the distance SMF which is equal for all rays at distance *r*. Since the location of plane A‐A on the z‐axis is of no influence on the resulting amplitude at *z*, we can eliminate $L_{0}$ from the equation. The cos(Θ) relation results from the geometry of a perfect parabolic mirror surface. The derivation of cos(Θ) and the definition of *L* can be found in Supplemental Document, Section A.

Adding up amplitudes from all sources by integrating over the parabolic mirror surface from r=0, till $r=R_{T}\;$ will then lead to an expression for the resulting intensity at location *z* on the optical axis. A source M on the parabolic mirror at a distance r has an amplitude E(r) defined by the Gaussian beam intensity profile I(r) according to $I\left(r\right)=I_{0}\;exp\left[-2{\left(r/R_{e}\right)}^{2}\right]$ and thus the amplitude E(r) becomes:

(11) $$E\left(r\right)=E_{o}\;exp\left[-{\left(r/R_{e}\right)}^{2}\right],$$

with $E\left(r\right)=\sqrt{I(r)}\;$ and $E_{o}=\sqrt{I_{o}}\;$.

According to the Huygens–Fresnel principle, we can describe an incoming ray at distance *r* reaching *z* on the optical axis via M by

(12) $$E\left(r,z,t\right)=E\left(r\right)\sin\left(\varphi \left(z,r\right)+\omega t\right),$$

where E(r,z,t) is the time dependent amplitude at location z for a ray at distance r, with ωt the time‐phase‐dependency. The spatial phase dependency, φ(z,r), is described by:

(13) $$\varphi \left(z,r\right)=k\left(L-L_{0}\right)=zk\;cos\left(\Theta \right),$$

where $k=\frac{2\pi n}{\lambda_{o}}\;$ with $\lambda_{0}$ the wavelength of light in vacuum. The resulting amplitude at location z is obtained after integration over all rays from the parabolic surface. This surface is limited however by the truncation radius *R*_T_. Thus when integrating over small ring segments with radius r and width dr the resulting amplitude at location *z* is found by the integral:

(14) $$\begin{array}{ccc} E\;\left(z,t\right) & = & \int_{0}^{r=R_{T}}E\left(r,z,t\right)2\pi rdr\\ & = & \;\pi R_{e}^{2}\int_{0}^{r=R_{T}}E\left(r,z,t\right)d{\left(r/R_{e}\right)}^{2}. \end{array}$$

After filling in E(r,z,t) (Equation 12) and φ(z,r) (Equation 13), Equation (14) becomes:

(15) $$\begin{array}{ccc} E\;\left(z,t\right) & = & \;\pi R_{e}^{2}E_{o}\int_{0}^{r=R_{T}}\exp\left[-{\left(r/R_{e}\right)}^{2}\right]\sin\left(zk\cos\left(\Theta \right)+\omega t\right)\\ & & \times \;d{\left(r/R_{e}\right)}^{2}, \end{array}$$

with E(z,t) the amplitude at *z* and time *t*, and $E_{0}$ the centre‐amplitude of the incoming Gaussian beam. The diffraction integral (Equation 15) is valid for all possible values of truncation, 0 < *T* < ∞ and *F#*_T_ > 0 (0…∞), with the consequence that this equation is valid for both the paraxial and non‐paraxial regime.

Equation (15) can be solved by numerical integration, which we have used to check the analytical solution that will be derived in the next section. The parabolic mirror model for the calculation of the extent of the focal region holds generally and therefore also for objectives and is appropriate for the situation in microscopy. The merit of this model is that it holds for any degree of truncation by the back‐aperture of the focusing optics.

## 5 PARAXIAL ANALYTICAL SOLUTION OF EQUATION (15)

The integral of Equation (15) can be solved when the paraxial approximation f≫r is invoked. The term cos(Θ) can be written as follows:

(16) cosΘ=4f2−r24f2+r2≈1−2r/Re22f2fReRe2.

Inserting this in Equation (15) and using the classical definition of the Rayleigh length $\;Z_{r}=\frac{2f^{2}}{kR_{e}^{2}}\;$, we obtain:

(17) $$\begin{array}{ccc} E\left(z,t\right) & = & \;\pi \;{R_{e}}^{2}E_{o}\int_{0}^{r=R_{T}}\exp\left[-{\left(r/R_{e}\right)}^{2}\right]\\ & & \times \;sin\left[zk+\omega t-\frac{z}{Z_{r}}{\left(r/R_{e}\right)}^{2}\right]d{\left(r/R_{e}\right)}^{2}. \end{array}$$

This integral can be solved (see Supplemental Document, Section B, for details) and results in the following expression for the intensity distribution along the *z*‐axis:

(18) Iz=πRe2Eo2exp−2−2T2T2−2exp−1−1T2T2coszZrT2+11+zzZrZr2,

with I(z) the intensity at location *z* and *T* the truncation value defined as in Equation (6).

Truncation, by itself, will lower the intensity in the focal region and also at location *z* = 0. To determine the resolution, the intensity, I(z) will be normalised to the intensity at the centre of the focus. This normalised intensity will be written as $I_{zn}\left(z\right)$. Since, the intensity in the focal plane at *z* = 0 is:

(19) Iz=0=πRe2Eo21−exp−1−1T2T22;

the normalised intensity $I_{zn}\left(z\right)$, after some algebra, becomes:

(20) Iznz=11+zzZrZr21+4·sin2zz2ZrT22ZrT2exp−1−12T22T2−exp112T22T22

This expression is consistent with the classical Rayleigh length definition as can be checked by inserting $z=Z_{r}\;$ and T=0 as for a non‐truncated Gaussian beam:

Iznz=Zr=11+zzZrZr2=12.

As expected from Equation (5), Equation (20) gives us the full intensity profile along the optical axis, but now explicitly including the truncation factor *T* (Equation 20) makes it possible to define any optical resolution definition (*z*), at any relative intensity level $I_{\mathrm{zn}}$(z), including the common choices FWHM, that is, $I_{\mathrm{zn}}$= 1/2 and the $I_{\mathrm{zn}}=1/e^{2}$ criteria. It can be seen that the 1/*e* ^2^ criterium will lead to the solution: $z=Z_{r}\;\sqrt{e^{2}-1}$

## 6 RESULTS AND DISCUSSION

In the following three sections, Equation (20) will be applied to the following three situations: (1) planar illumination of the aperture, (2) effect of truncation by the aperture on the resolution and power loss of a Gaussian beam, (3) addition of an empirical term to extend the applicability of Equation (20) into the non‐paraxial regime.

### 6.1 Planar illumination in the paraxial approximation

The planar illumination with a truncated Gaussian beam is obtained in the limit of infinite truncation, that is, *T* = ∞. Equation (20) for this limit becomes:

(21) $$I_{\mathrm{zn}}\left(z\right)=\frac{\sin^{2}\left(\frac{z}{2Z_{r}T^{2}}\right)}{{\left(\frac{z}{2Z_{r}T^{2}}\right)}^{2}}={\text{sinc}}^{2}\left(\frac{z}{2Z_{r}T^{2}}\right).$$

The detailed steps towards this result can be found in the Supplemental Document, Section C.

We analyse here this expression in depth, as one may be surprised by the presence of the term $\frac{1}{T^{2}}$. However, since the argument $\frac{z}{2Z_{r}T^{2}}$ with $Z_{r}$ defined by Equation (8), Equation (21) is independent of the truncation parameter T. The argument $\frac{z}{2Z_{r}T^{2}}$ becomes:

(22) $$\frac{z}{2Z_{r}T^{2}}=z\frac{\pi \;n}{8\lambda_{o}F{{\#}_{T}}^{2}}.$$

In the paraxial limit and by applying Equation (7), the *F#*_T_ becomes $F{\#}_{T}=\frac{n}{2NA_{T}}\;$. Inserting this in the argument $\frac{z}{2Z_{r}T^{2}}$, the argument becomes:

(23) $$\frac{z}{2Z_{r}T^{2}}=z\frac{\pi NA^{2}}{2n\lambda_{o}}.$$

With the definition for the standardised optical coordinate $u=z\frac{2\pi NA^{2}}{n\lambda_{o}}$, it can be noticed that Equation (21) is identical to the result from diffraction theory $I\left(0,u\right)∼\sin c^{2}\left(\frac{u}{4}\right)$,^7^ with the understanding that Equation (21) is the result of consistent reasoning with the F# and the inclusion of the truncation parameter, T.

#### 6.1.1 Other typical results from the paraxial planar illumination

The FWHM condition $\left(\;I_{\mathrm{zn}}=\frac{1}{2}\;\right)$ at Equation (21) leads to the solution

$$z=Z_{r}\cdot T^{2}\cdot 2.78312.$$

In this way we can, with the use of Equation (6), define $Z_{r\infty }=\frac{4\lambda_{0}F{{\#}_{T}}^{2}}{\pi n}\;\;2.78312,$ where $Z_{r\infty }$ represents the distance away from the focal centre, to where the intensity has dropped to half the intensity of the centre. This is the same definition as for the classical Rayleigh length (*T* = 0), but now for the Planar illumination situation (*T* = ∞).

The minima $I_{\mathrm{zn}}$= 0 (also at *T* = ∞) can likewise be found when the sine term in the sin*c* function (Equation 21) becomes zero, thus when $\frac{z}{2Z_{r}T^{2}}=N\pi$ (with *N* = 1, 2, 3, … the locations of the zero's in the sin*c* pattern) namely: $Z_{0}=\frac{8N\lambda_{0}F{{\#}_{T}}^{2}}{n}=2.2576\cdot N\cdot Z_{r\infty }$ This outcome is in exact agreement with the calculated numerical outcome from Equation (15) for the Planar illumination condition, (*T* = ∞).

### 6.2 Effect of truncation of a Gaussian beam

The second example to which we will apply the result of Equation (20) is to show the effect of truncation on power loss and resolution. The numerator (exp[−1/−12T22T2]−exp[1/12T22T2])2 in Equation (20) is a power loss term due to truncation of the Gaussian beam, since Ploss=exp[−2/−2T2T2] it can also be written as $\left(\sqrt{P_{\mathrm{loss}}}-2+\sqrt{1/P_{\mathrm{loss}}}\right)$.

**FIGURE 5:**
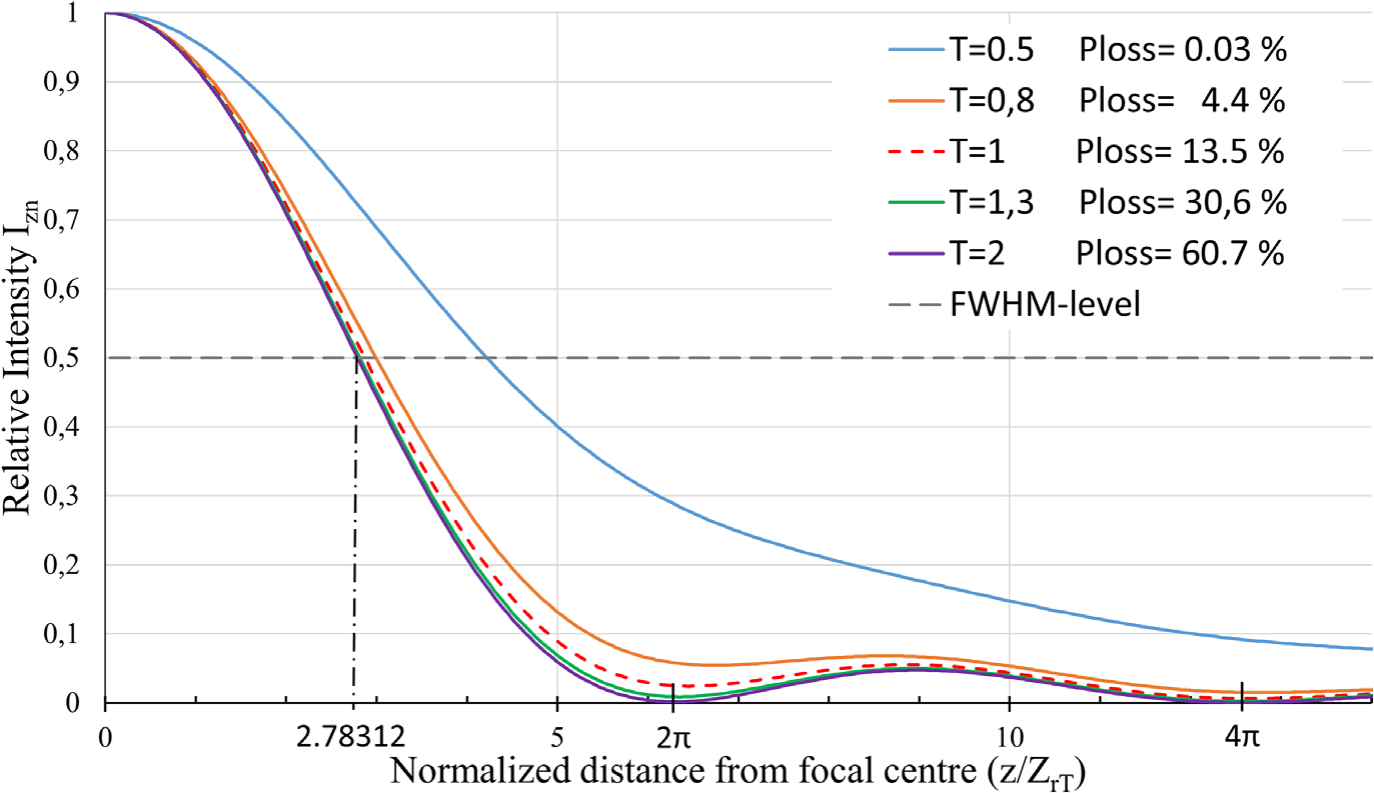
Intensity profile along the normalised *z* axis at different truncation values and its corresponding power loss. As an example of a choice of the resolution, the FWHM level has been drawn. It is to be noted that as the truncation value becomes smaller, the resolution decreases.

With the relationship between $I_{\mathrm{zn}}$, z, $Z_{r},$ and *T* of Equation (20), the trade‐off between the power loss and the resolution can be made. This is illustrated in Figure 5.

**FIGURE 6:**
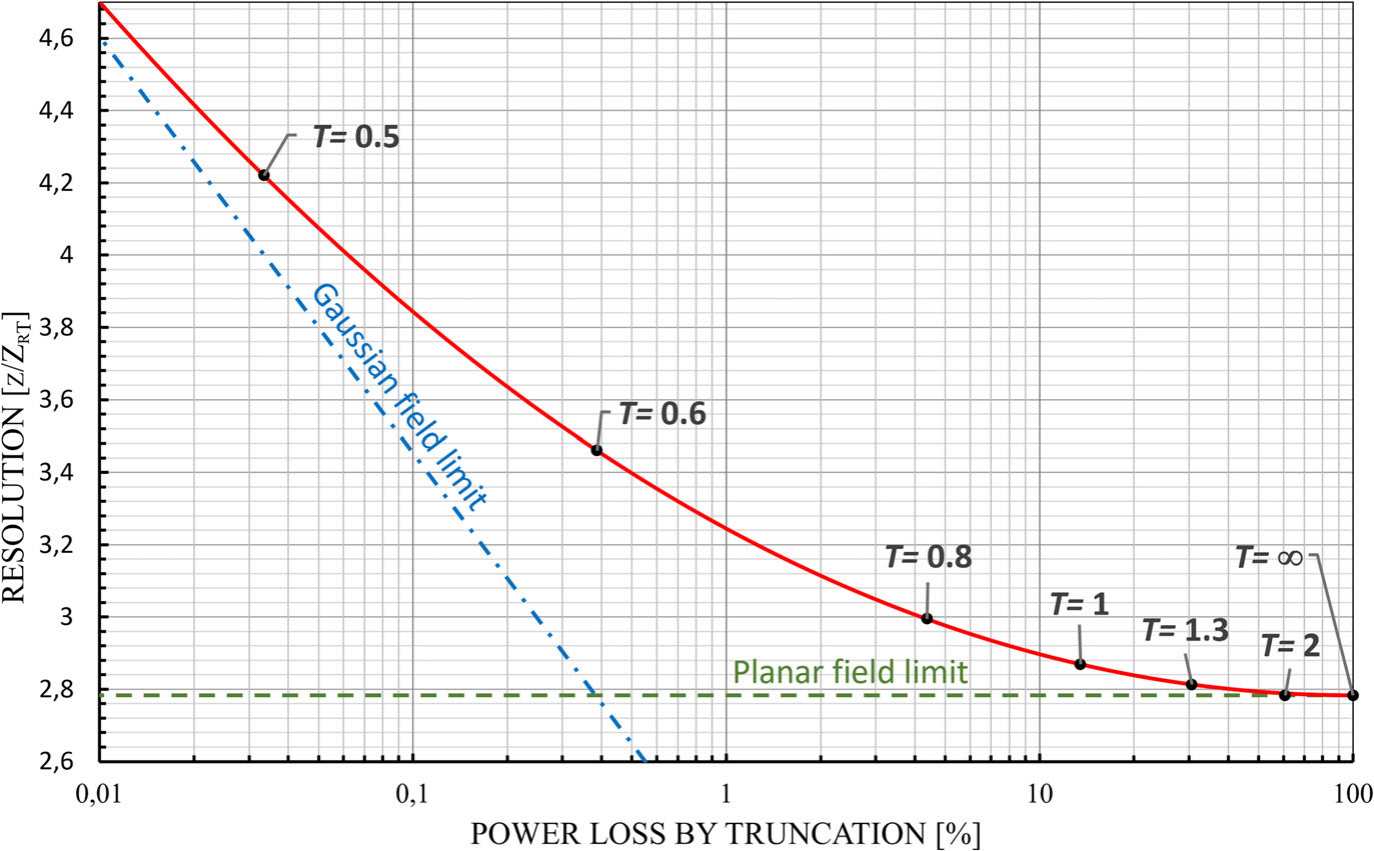
Resolution based on the FWHM condition ($I_{\mathrm{zn}}=\frac{1}{2}\;$) and represented by the value $\frac{z}{Z_{\mathrm{rT}}}$ as a function of Power loss by truncation.

In Figure 5, the normalised intensity profiles are plotted against the normalised propagation along the optical axis for several values of the truncation factor, each associated with the corresponding power loss. The *z*‐axis is normalised on $Z_{\mathrm{rT}}$, where $Z_{\mathrm{rT}}=Z_{r}\;T^{2}$. This normalisation makes the outcome independent on the chosen f‐number *F#*_T_ or *NA*. From Figure 5, it can be noticed that for *T* = 1 and $z/Z_{\mathrm{rT}}=2\pi$, the first Airy minimum has become clearly larger than ‘zero’. This treatment enables a rational approach to the trade‐off between resolution and power loss. The trade‐off between resolution and power loss as a result of truncation is concisely shown in Figure 6, where the resolution is plotted against the power loss as a result of truncation. The resolution criterion is chosen as the FWHM condition (see Figure 2). That is, *I*_zn_(*z*) *=* 1/2 in Equation (20). When the power loss is approaching zero, which means that *T* is approaching zero, the light distribution in the lateral focal plane approaches the Gaussian field distribution. When *T*→∞ the power loss is approaching 100%, and therefore, the axial light distribution is approaching the sin*c* function (Equation 21) and thus the limiting value of 2.78312 is achieved for $\frac{z}{Z_{\mathrm{rT}}}$ (see Section 6.1.1).

By rewriting Equation (20), a position *z* can be determined at any chosen value $I_{\mathrm{zn}}$ (Equation 24). We notice that *z* is not a simple function of $I_{\mathrm{zn}}$, *Z*_r_, and T as *z* is found on the left and right of the equal sign in Equation (20). However, Equation (24) can be iteratively solved for *z* in a rapidly converging process, as shown in Supplemental Document, Section D.

(24) z=Zr1Izn−1+4·sin2z2ZrT2Iznexp−1−12T22T2−exp112T22T22.

This result contains the classical Rayleigh length definition for an untruncated Gaussian beam. The value can be found by setting *T* = 0 and $I_{\mathrm{zn}}$= 1/2 in Equation (24) and the original FWHM definition $z=Z_{r}\;$ is obtained, as expected.

By adding a truncation‐dependent term to the classical Rayleigh length definition (Equation 24), the power‐loss and resolution for truncation values between 0≤T≤∞ are correctly taken into account.

It may be noted that Equation (20) gives a full intensity profile along the optical axis and a precise relation is obtained between truncation, power loss and resolution, as shown in Figures 5 and 6. As an example the FWHM resolution criterion was shown in Figure 5, but it is apparent that other choices for defining the resolution may be made.

The result of the analytical expressions (Equations 20 and 24) is in exact agreement with the numerical calculations (result not shown) of Equation (15) over the entire truncation range 0≤T≤∞ and the entire intensity range $0\leq I_{\mathrm{zn}}\leq 1$, under paraxial conditions.

### 6.3 Inclusion of the non‐paraxial regime

Equation (24) can be extended to encompass the entire range of possible f‐number (*F#*) values and therefore to include high *NA* optical elements for which the paraxial approximation ($R_{T}\ll 2f$), see Figure 4, does not hold.

To include the non‐paraxial regime, Equation (24) is extended by multiplication with a single factor, *C*_np_, which contains the f‐number, *F*#_T_, and the truncation factor, *T*, as follows:

(25) $$C_{\mathrm{np}}=1+\frac{1}{15.4F{{\#}_{T}}^{2}}\exp\left[\frac{-1}{10.4T^{2.5}}\right].$$

This factor *C*_np_ is empirically determined by comparing the outcome of the analytical expression (Equation 24) with the exact numerical outcome of the integral from Equation (15). More explanation about how we came to the expression for *C*_np_ and its dependency on truncation *T* and f‐number $F{\#}_{T}$ can be found in Supplemental Document, Section E. Equation (25) is only valid for the common FWHM resolution criterion (Equation 5), that is, $I_{\mathrm{zn}}=1/2\;$ and other expressions can be determined for other resolution criteria. When inserting *C*_np_ in Equation (24), Equation (26) is obtained:

(26) ZFWHM=ZrCnp1+8·sin2ZFWHM2ZrCnpT2exp−1−12T22T2−exp112T22T22.

Again $Z_{\mathrm{FWHM}}$ is present on both sides of the equation and its value can be found after iteration with the procedure in Supplemental Document, Section D. Equation (26) accurately describes the intensity along the optical axis for high *NA* illumination and collection, with the understanding that Equation (26) is only valid for T≥0.5 and $F{\#}_{T}\geq 0.34$, which, in terms of *NA*, equates to an *NA* limitation of NA<0.95n. Equation (26) thus covers correctly the entire range of high *NA* illumination optics, which is common in high resolution optical microscopy. The outcome of this *Z*_FWHM_ definition (Equation 26) is compared with the numerical outcome of the exact integral (Equation 15) and shows that Equation (26) has an error of less than 0.4% over the range of NA as stated above. The practical importance of the empirically determined factor *C*_np_ becomes clear if *C*_np_ is set to ‘1’. The error in $Z_{\mathrm{FWHM}}$ increases to 36% in that case.

We have investigated, whether the multiplier *C*_np_ is related to the *M* ^2^ beam quality.^17^, ^18^ We conclude that, although there are similarities, a precise relationship would only exist if all the truncation dependencies in Equation (26) were confined in the definition of factor *C*_np_. We conclude therefore that the factor *C*_np_ bears no straightforward relationship with the *M* ^2^ beam quality factor.

## 7 CONCLUSION

We presented a new expression (Equation 20) for the description of the intensity of the light‐field along the optical axis after a focusing element with the inclusion of truncation. The derivation of this analytical expression is based on a systematic implementation of F# instead of NA for reasons that the F# enables the inclusion of high angle rays up to 180°. The expression includes all relevant experimental parameters, such as the truncation factor, the power loss due to truncation, the refractive index, the *F#* of the focusing element and the wavelength of light. The validity of the expression was exemplified for planar illumination and Gaussian beam illumination at any truncation value between 0 and infinity.

The expression describes the effect on the Rayleigh length for any degree of truncation, where up to now formulas were presented for limiting cases only, namely small truncation, approaching a Gaussian beam illumination, large truncation, approaching a planar illumination or truncation around *T* = 1. Rewriting Equation (20) into a version in which *z* becomes a function of $\left\{I_{\mathrm{zn}},T,Z_{r},\right\}$ (Equation 24), we have extended the classical Rayleigh length definition (Equation 5), with a new, single closed expression (Equation 20), that includes the entire range of possible truncation factors, and that is valid under the paraxial condition. We propose that the analytical expressions, Equations (20) and (24) are useful in the field of microscopy and spectroscopy with Gaussian wavefronts, and is a useful addition to the theory regarding Gaussian beam optics.

The applicability can be extended to the non‐paraxial regime by adding an empirical factor *C*_np_ to Equation (21), for *I*_zn_(*z*) fixed to ½, a FWHM resolution outcome is obtained (Equation 26). The limitation of Equation (26) by T≥0.5 and $F{\#}_{T}\geq 0.34$ is no limitation to the practical use, since it still covers the entire practical field of microscopy. The error of Equation (26) in the non‐paraxial regime is less than 0.4% achieved for high NA = 0.95.

## CONFLICT OF INTEREST STATEMENT

The authors declare no conflicts of interest.

## Supporting information



Supporting information

## References

[1] Enciso‐Martinez, A. , van der Pol, E. , Lenferink, A. T. M. , Terstappen, L. W. M. M. , van Leeuwen, T. G. , & Otto, C. (2020). Synchronized Rayleigh and Raman scattering for the characterization of single optically trapped extracellular vesicles. Nanomedicine, Nanotechnology, Biology and Medicine, 24, 102109. 31669420 10.1016/j.nano.2019.102109

[2] Gillibert, R. , Balakrishnan, G. , Deshoules, Q. , Tardivel, M. , Magazzu, A. , Donato, M. G. , Marago, O. M. , de, L. a. , Chapelle, M. L. , Colas, F. , Lagarde, F. , & Gucciardi, P. G. (2019). Raman tweezers for small microplastics and nanoplastics identification in seawater. Environmental Science & Technology, 53(15), 9003–9013. 10.1021/acs.est.9b03105 31259538

[3] Horváth, Z. L. , & Bor, Z. (2003). Focusing of truncated Gaussian beams. Optics Communications, 222, 51–68.

[4] Agrawal, G. P. , & Pattanayak, D. N. (1979). Gaussian beam propagation beyond the paraxial approximation. Journal Optical Society of America, 69, 575–578.

[5] Sheppard, C. J. R. (1988). Depth of field in optical microscopy. Journal of Microscopy, 149, 73–75.

[6] Drège, E. M. , Skinner, N. G. , & Byrne, D. M. (2000). Analytical far‐field divergence angle of a truncated Gaussian beam. Applied Optics, 39(27), 4918–4925. 10.1364/ao.39.004918 18350085

[7] Urey, H. (2004). Spot size, depth‐of‐focus, and diffraction ring intensity formulas for truncated Gaussian beams. Applied Optics, 43(3), 620–625. 10.1364/ao.43.0006209 14765922

[8] Schell, R. G. , & Tyras, G. (1971). Irradiance from an aperture with a truncated‐Gaussian field distribution. Journal of the Optical Society of America, 61, 31–35.

[9] Dickson, L. D. (1970). Characteristics of a propagating Gaussian beam. Applied Optics, 9, 1854–1861. 20094152 10.1364/AO.9.001854

[10] Li, Y. (1988). Variations of the axial intensity pattern formed by a focused truncated Gaussian beam. Optics Communication, 68, 324–328.

[11] Virendra, N. , & Mahajan, N. V. (1986). Uniform versus Gaussian beams: A comparison of the effects of diffraction, obscuration, and aberrations. Journal of the Optical Society of America A: Optics and Image Science, and Vision , 3(4).

[12] Siegman, A. E. (1986). Lasers. University Science, Chapter 16.

[13] Damask, J. N. (2004). Polarization optics in telecommunications (pp. 219–224). Springer.

[14] Siegman, A. E. (1986). Lasers. University Science, Chapter 17, 668.

[15] Siegman, A. E. (1986). Lasers. University Science, Chapter 17, 666.

[16] Lieb, M. A. , & Meixner, A. J. (2001). A high numerical aperture parabolic mirror as imaging device for confocal microscopy. Optics Express, 8(7), 458–474. 19417842 10.1364/oe.8.000458

[17] Siegman, A. E. (1998). How to (maybe) measure laser beam quality. In M. Dowley (Ed.), DPSS (diode pumped solid state) lasers: Applications and issues (Vol. 17 of OSA Trends in Optics and Photonics). Optica Publishing Group.

[18] Mei, Z. , & Zhao, D. (2005). Approximate method for the generalized M2 factor of rotationally symmetric hard‐edged diffracted flattened Gaussian beams. Applied Optics, 44(8), 1381–1386. 10.1364/ao.44.001381 15796235

