# Supplementary material for: Analytical solution of the classical Rayleigh length definition, including truncation at arbitrary values
Source: J Microsc. 2025 Jul 31;300(1):124–33. doi: 10.1111/jmi.70016 (PMC12459428; doi:10.1111/jmi.70016)
Supplement: Supplementary file 1 — Supporting information [file JMI-300-124-s001.pdf]

Analytical solution of the classical Rayleigh length definition, including truncation at arbitrary values (Supplemental document)

AUFRIED LENFERINK | CEES OTTO

Department of Bio Engineering and Technology, Technical Medical Centre, Faculty of Science & Technology, University of Twente, 7522NH Enschede, The Netherlands

Correspondence

Aufrid T.M. Lenferink, University of Twente, Hallenweg 23, 7522NH Enschede, The Netherlands
Email: a.t.m.lenferink@utwente.nl Tel:+31 53 4893099

Section A

Derivation of $\cos(\theta)$ in Equation 10 of the main document

From Figure 4. of the main document the following drawing Figure S1 was made:

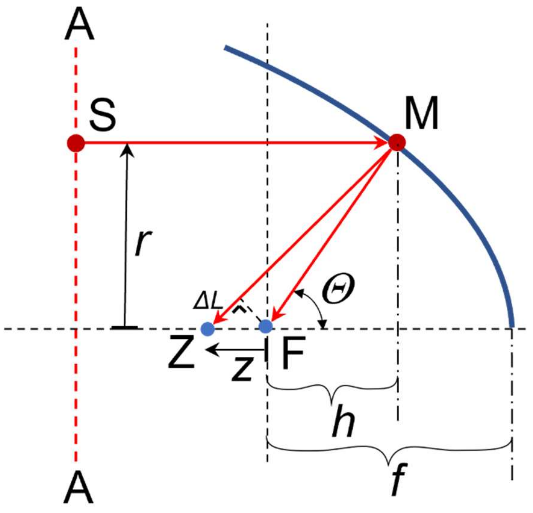

FIGURE S1. Path length L definition.

The path length L is defined as $L = \mathrm{SMZ} = \mathrm{SMF} + \Delta L$. The plane A-A represents the wavefront of an incoming gaussian beam. All rays at any distance *r,* and parallel to the optical (z-) axis, will reach a point M on the parabolic mirror. A spherical wave from M is reaching a location Z at distance z from focal point F. Due to the use of the parabolic reflecting interface, all distances SMF are equal for all values of *r*. The distance ΔL can be defined as $\Delta L = z\cos(\theta)$. This is a common approximation when f >> z, and which complies with the present situation where f is of the order of millimeter and z is of the order of micrometer.

The term $\cos(\theta)$ can be expressed in r. For this the equation for the parabolic surface is written as $r^2 = 4f(f-h)$ and thus h is defined as:

$$h = f\left(1 - \frac{r^2}{4f^2}\right)$$

With f the focal distance of the parabolic mirror

The angle θ is defined by $\cos(\theta) = \frac{h}{\sqrt{h^2+r^2}}$

And thus: $h^2 + r^2 = f^2\left(1 - \frac{r^2}{4f^2}\right)^2 + r^2 = f^2\left(1 + \frac{r^2}{4f^2}\right)^2$

And therefore $\cos(\theta) = \frac{1-\frac{r^2}{4f^2}}{1+\frac{r^2}{4f^2}} = \frac{4f^2-r^2}{4f^2+r^2}$

Section B

Solution of the diffraction integral of Equation 17 from the main document.

$$E(z,t) = \pi\, R_e^{\,2}\, \hat{E}_o \int_0^{R_T} \exp\left[-\left({}^{r}/_{R_e}\right)^2\right] \sin\left[zk + \omega t - \frac{z}{Z_r}\left({}^{r}/_{R_e}\right)^2\right] \mathrm{d}\left({}^{r}/_{R_e}\right)^2$$

This expression can be simplified by making the following substitutions:

$$Q = \pi R_e^{\,2} \hat{E}_o, \quad B = z/Z_r, \quad X = \left({}^{r}/_{R_e}\right)^2 \text{ and } \quad C = z\,k + \omega t$$

When applied, this also changes the integration limit r=R_T to X=$1/T^2$ since T= R_e/R_T we obtain:

$$E(z,t) = Q \int_0^{1/T^2} \exp\,[-X] \sin\,(C - BX)\mathrm{d}X$$

To solve this integral, we first substitute: $Y = C - BX$ and: $\mathrm{d}X = -\mathrm{d}Y/B$ to obtain:

$$E(z,t) = -\frac{Q}{B} \exp[-{}^{C}/_{B}] \int_{Y=C}^{Y=C-B/T^2} \exp\,[{}^{Y}/_{B}] \sin\,(Y)\mathrm{d}Y$$

This integral can be solved,[S1] and the solution is:

$$E(z,t) = \frac{Q}{B} \exp[-{}^{C}/_{B}] \left[\frac{\exp[{}^{Y}/_{B}]}{1 + {}^{1}/_{B^2}} \left\{\frac{\sin(Y)}{-B} + \cos(Y)\right\}\right]_{Y=C}^{Y=C-B/T^2}$$

The addition of a sine and a cosine with different amplitudes leads to:[S2]

$$E(z,t) = Q\exp[-{}^{C}/_{B}] \left[\frac{\exp[{}^{Y}/_{B}]}{\sqrt{1 + B^2}} \{\sin(\alpha + Y)\}\right]_{Y=C}^{Y=C-B/T^2}$$

With $\tan \alpha = -B$

Subsequently, we perform the inverse substitution: $Y = C - BX$ to obtain:

$$E(z,t) = \frac{Q}{\sqrt{1 + B^2}} [\exp[-X] \sin(\alpha + C - BX)]_{x=0}^{x=1/T^2}$$

After inserting the boundaries, we obtain:

$$E(z,t) = \frac{Q}{\sqrt{1 + B^2}} \left[\exp\left[-{}^{1}/_{T^2}\right] \sin\left(\alpha + C - \frac{B}{T^2}\right) - \sin(\alpha + C)\right]$$

The addition of two sine terms with a phase difference $\left(-\frac{B}{T^2}\right)$ and different amplitudes leads to:[S2]

$$E(z,t) = Q\frac{\sqrt{\exp\left[-{}^{2}/_{T^2}\right] - 2\exp\left[-{}^{1}/_{T^2}\right] \cos\left(\frac{B}{T^2}\right) + 1}}{\sqrt{1 + B^2}} \sin(\beta + C)$$

with β the new phase. Since we are only interested in the resulting amplitude $\hat{E}(z)$ at location z and not its time dependency $E(z,t)$, the sine term; $\sin(\beta + C\,)$can be replaced by "1". The intensity at location z then becomes:

$$I(z) = \hat{E}(z)^2 = \frac{Q^2\left\{\exp\left[{-2}/{T^2}\right] - 2\exp\left[{-1}/{T^2}\right]\cos\left(\frac{z}{Z_r T^2}\right) + 1\right\}}{1 + \left({z}/{Z_r}\right)^2}$$

Section C

Solving Equation (20) from the main document for planar illumination.

When T is approaching infinity, each exponential term in the denominator of expression Equation (S1) will approach "1" or according to series in math; $\exp\left[{-1}/{2T^2}\right]$ will approach to $1 - {1}/{2T^2}$ and $\exp\left[{1}/{2T^2}\right]$ will approach to $1 + {1}/{2T^2}$.

$$I_{zn}(\mathrm{z}) = \frac{1}{1+\left({z}/{Z_r}\right)^2}\left[1 + \frac{4\sin^2\left({z}/{2Z_r T^2}\right)}{\left(\exp\left[{-1}/{2T^2}\right] - \exp\left[{1}/{2T^2}\right]\right)^2}\right] \quad \text{(S1)}$$

Subtraction and raised to the power 2, the denominator will approach to ${1}/{T^4}$
This results in:

$$I_{zn}(z) = \frac{1}{1+\left({z}/{Z_r}\right)^2}\left[1 + \frac{4\sin^2\left({z}/{2Z_r T^2}\right)}{{1}/{T^4}}\right] = \frac{1}{1+\left({z}/{Z_r}\right)^2}\left[\frac{4\sin^2\left({z}/{2Z_r T^2}\right)}{{1}/{T^4}}\right]$$

Next it can be concluded that when $T = \infty$, Zr becomes small (see definition from Eq. (6) in the main document). This means the term "1" in the denominator becomes insignificant, and thus:

$$I_{zn}(z) = \frac{1}{\left({z}/{Z_r}\right)^2}\left[\frac{4\sin^2\left({z}/{2 \cdot Z_r \cdot T^2}\right)}{{1}/{T^4}}\right] = \frac{\sin^2\left({z}/{2Z_r T^2}\right)}{\left({z}/{2Z_r T^2}\right)^2}$$

Section D

The following approach to quickly, iteratively solve Equation (24) of the main document is to have a variable $H = \frac{z}{Z_r T^2}$ on both sides of the equation. For this, Equation (24) will be divided by $Z_r T^2$ on both sides of the equal sign.

$$H' = \frac{1}{T^2}\sqrt{\frac{1}{I_{zn}} - 1 + \frac{4\cdot\sin^2\left({H}/{2}\right)}{I_{zn}\left(\exp\left[{-1}/{2T^2}\right] - \mathrm{ex}\ \left[{1}/{2T^2}\right]\right)^2}}$$

Now the iteration starts with $H = 1 + 1/T^2$

Next calculate the new H' with the formula above

Subsequently calculate the new H with $H = H' + (H' - H)I_{zn}$

Again use the formula to calculate the next H'

Etc. until the $H' \approx H$ to the desired accuracy.

This procedure enables rapid convergence of the iteration.

After H is found, z can be found with $z = HZ_r T^2$

Section E

Explanation of the approximation of the expression for C_{np} and its dependency on truncation T and f-number $F\#_T$.

Necessary restrictions for the expression are:

1. Not conflicting with the analytical paraxial expression Equation (24) of the main document, meaning: when $F\#_T \to \infty$ than $C_{np} \to 1$
2. F-number range is restricted to the range for modern objectives used in practical microscopy $F\#_T > 0.34$
3. The un-truncated situation $T \to 0$ should lead to $C_{np} \to 1$. This situation leads to the paraxial condition since $F\#_T$ cannot be zero (restriction #2).
4. Only valid for the FWHM condition ($I_{\text{zn}} = 1/2$).

Observing the 4 restrictions from above a first guess would be $C_{np} = 1 + G$, with G a function of T and $F\#_T$.

How would the term *G* depend on T?

This was tested at a fixed-midrange-value for the f-number, $F\#_T = 1$. To minimize the difference between the outcome of the analytical expression (with $C_{np} = 1 + G$) and the numerical outcome. G was fitted with $\text{A} \cdot exp[-1/\text{B}T^c]$ with A, B and C constants.

How would the term *G* depend on $F\#_T$?

This was tested at a fixed-midrange-value for truncation, $T = 1$. To minimize the difference between the outcome of the analytical expression (with $C_{np} = 1 + G$) and the numerical outcome. G was fitted with $\frac{1}{\text{D}\cdot F\#_{\text{T}}{}^2}$ with D a constant.

Combining both results by substituting for A, $\frac{1}{\text{D}\cdot F\#_{\text{T}}{}^2}$ leads to the expression:

$$C_{\text{np}} = 1 + \frac{1}{\text{D} \cdot F\#_T{}^2} exp\left[\frac{-1}{\text{B} \cdot T^{\text{C}}}\right]$$

After establishing the coarse expression for the dependencies on T and $F\#_T$, it is noticed that this expression is not conflicting with any of the restrictions mentioned above. This expression is now used for a fit that optimizes all parameters D, B and C for a best possible fit of the analytical expression and the numerical outcome.

The optimized values from the fit were: D=15.4 , B=10.4 and C=2.5.

With these parameter values, the error between the outcome of the analytical expression and the numerical outcome of the exact diffraction integral was less than 0.4%.

References

S1. Gradshteyn and Ryzhik's Table of Integrals, Series, and Products, Daniel Zwillinger and Victor Moll (eds.), Seventh edition (2007), Chapter 2.663, 228. ISBN-11: 0-12-373637-4

S2. Hecht, Optics, 4th Edition, Chap 7.1, 286, ISBN:0-321-18878-0
